# Cooking and Catering in Mental Hospitals

**Published:** 1913-10-11

**Authors:** J. Francis Dixon

**Affiliations:** Medical Superintendent, Borough Mental Hospital, Leicester


### COOKING AND CATERING IN MENTAL HOSPITALS.

### The Cause and Cure of "Institutional Cooking."

By J. FRANCIS DIXON, M.D., Medical Superintendent, Borough Mental Hospital, Leicester.

One of the commonest complaints in large institutions and particularly in mental hospitals is about the food, and to satisfy a thousand people with the same dish at a minimum cost is no easy task. The resources of the central kitchen and the strength of its staff do not admit of the preparation of a great variety of diets at the same time. Decentralisation may be a cure for "institution" cooking, but is probably a very expensive one.

To what is due the stigma attached to "institution" cooking? Largely, I should say, to lack of appreciation of the fact that even *plain* cooking is more or less of a fine art, and that it requires no small amount of intelligence, skill, and knowledge to treat even everyday foodstuffs in just the proper manner. As a nation we are notoriously ignorant in this matter, and until cooks become the properly trained and highly paid functionaries their position demands, so long will the devil get the credit of sending them to us. But even the best of cooks finds it difficult to make bricks without straw. The quality of the meat, vegetables, etc., delivered into her hands is often far from the best. In some mental hospitals, which have land enough, the milk, etc., is supplied from the farm, and the dried-off milch cows slaughtered; thus cow-beef comes largely into the diet. Old ewe mutton also is not uncommon. In many institutions, owing to the soaring price of the home-grown article, Argentine beef has been introduced with varying results. Quite consistent, however, is the satisfaction given by New Zealand mutton. New Zealand stands alone for quality in both its tinned and frozen meat, and should not, as is generally the case, be lumped up with Australia. There is about as much difference between the sheep pastures of New Zealand and Australia as there is between those of England and Spain.

There is, of course, no meat in the world to equal well-bred, well-fed English meat, and there is no supply more difficult to keep at a high standard than that of meat. Argentine and other foreign meats tend to deteriorate in quality owing to the nature of the cattle feed. This can be counteracted only by the frequent introduction of the finest English blood, and has been done to such good purpose of late years that Argentine chilled beef (not frozen) of the best quality is in fact superior to a great deal of second-class English beef. Chilled beef is difficult to handle, owing to its bad keeping qualities, and mental hospitals situated inconveniently for quick transit are debarred its use. Any delay in delivery and it becomes tainted. Frozen beef if bought properly is a sound food, but rarely possesses the distinctive character that belongs to English-fed or chilled beef. The practice followed here is to buy home-grown beef in what is known in the district as "beds and rounds" and "legs and shins." This has distinct advantages—namely, the round provides good roasting meat, and the bed excellent meat for salting. Another advantage of the bed and round is that the proportion of bone to meat is between 6 and 7 per cent. only. For soups just the quantity required of coarse meat can be purchased in the form of legs and shins. For the officers' mess joints at a fixed price are obtained.

This arrangement dispenses with the necessity of carrying a butcher on the staff, the cutting up of mutton (which of course is purchased in carcases) being easily done by the ordinary stores staff. The pigs we have always with us, and it is largely on the nature of their food that their health and the quality of the pork depend. Here, we sometimes pick up the thread of a vicious circle. Inferior meat badly cooked and rejected passes into the swill-tub: result—unhealthy pigs, bad pork, and the encouragement of cannibalistic tendencies. Besides, it is poor economy to feed pigs on food prepared for human consumption. Then, again, the fish provided is mostly of a coarse variety, but with the introduction of the fish frier it can readily be made most palatable. As to vegetables, it is frequently overlooked that some varieties are useless after reaching to a certain age and come out of the boiler in practically the same condition as they went in. Boiling has no effect on ligneous fibre. But let us suppose the cook cannot reasonably grumble about her materials—happy cook! How often do we see good meat done to a cinder, shrivelled to half its original weight and all its juice gone, just because evaporation is allowed to go on unchecked.

Is the ration allowance in terms of cooked meat free from bone or of raw meat as delivered from the butcher? In the former case such cooking is doubly bad owing to excessive waste. Dealing with large numbers in bulk it is perhaps the more convenient basis, but for the staff, having broken up the mess into groups at small tables as described in a previous article, it is found better to adopt the latter basis and so avoid hacking the meat about before serving. This entails labelling the various portions before cooking.

A point to remember about cooking green vegetables is that they require lots of water. If bulky, such as cabbage, the capacity of an ordinary equipment of boilers may be overtaxed. This difficulty is met by cooking such things as cabbage for one division of the hospital only at a time. Concerning cabbage—boiled bacon and cabbage is extremely popular providing you boil the bacon and the cabbage together. Green cabbage when cooked should be green; green peas when cooked should be green. Whether they are green or brown depends on a few pennyworth of bi-carbonate of soda.

The appearance of things has a great bearing on successful dieting and so has the smell. If a thing looks right and smells right, it usually tastes right. And now we will suppose we have our dinner ready to serve. Whether in a large dining hall or in the ward, general principles can be carried out. As far as possible all having the same diet should sit together. The plates should not be piled up with food thrown on anyhow. The reason this sometimes happens is no doubt to make a second helping unthinkable, thus saving time and trouble. But it is not good service. Fat and lean should be cut in proper proportions and of reasonable thickness. Where there are large numbers with a small staff and a half-hour time limit, individual attention must be got by grouping. This may be based on age, activity, temperament, habits, etc. An active old dement does not require the same amount or kind of food as an energetic young farm-worker. Epileptics and the excitable do best with little or no meat. People who cannot be taught decent table manners, and are a source of annoyance and discomfort to their less afflicted neighbours, should be separated from them. Patients on ordinary diet sometimes like a change for a time to milk and farinaceous diet, or vegetable diet, and can join a corresponding group.

Each ward has a diet board, on one side of which is marked daily the total number of patients in the ward, with the numbers on each diet; and on the other side a description of the set diets. These last consist of various quantities and combinations of milk, eggs, custard, farinaceous puddings, beef-tea, broth, bovril, and bread and butter. There is one comparatively costly diet for convalescents. There are no extras. The ordinary diet is served to nearly 70 per cent. of the patients and is made as varied as possible. If every Friday regularly brings fish along, we ring the changes by having it baked, boiled, or fried, while the herring in its various forms is "something different."

One of the most popular introductions into the dietary of mental hospitals in recent years is fried fish. Vinegar being used instead of the fish sauce issued with baked or steamed fish. Whilst fish sprinkled with flour and bread raspings looks better when served, that dipped in batter is more popular. By making dripping the frying medium a satisfactory means of using up this material is attained. The smelly cotton-seed oil commonly used, however, is cheaper and said to give better cooking results. The cost of installing the necessary cooking plant is comparatively small, but it is advisable to go much beyond the capacity recommended by the makers if the meal is to be properly rounded off with chipped potatoes. The output of a six-pan suite is about 120 lb. of potatoes in forty-five minutes.

The dinners provided for patients on vegetarian diet, contrary to what one might think, have given great satisfaction, and I append one or two of the more popular recipes. The vegetables, of course, have to be varied according to the season, but those below give a general idea.

As noted bread *ad lib.* is served with all dinners, and in order to save unnecessary waste it is cut in comparatively small pieces and given as required. Thus, very little finds its way into the swill-tub, and there is no stint. In the case of epileptics, special bread is prepared in which the chloride is replaced by the bromide salt. The difference is hardly noticeable and the specific effect quite satisfactory. I am much indebted to Mr. Sidney C. Leak, the clerk and steward of the mental hospital, for his very able assistance in working out these recipes and their cost.

### RECIPES.

### Tomato Broth.

*Scale per Gallon.*

1¼ lb. Turnips.
1 lb. Onions.
2 oz. Pea flour.
2 oz. Haricots.
2 oz. Lentils.
2 lb. Potatoes.
8 oz. Tomatoes.
Pepper, salt.
Average cost .43 pence per head.

### Vegetable Rissoles.

*Scale per Patient.*

1 oz. Onions.
1 oz. Dripping or minced fat bacon.
1 oz. Carrots.
1 oz. Lentils.
4 oz. Bread crumbs.
½ oz. Parsley.
½ oz. Cheese.
1 oz. Milk.
1 oz. Flour.
Pepper, salt.
Average cost 1.21 pence per head.

### Curry. *Scale per Patient.*

¾ oz. Cheese.

¾ oz. Rice.

1 oz. Onions.

¾ oz. Pearl barley.

1/64 oz. Curry powder.

⅛ oz. Anchovy sauce.

Pepper, salt.

Average cost .55 pence per head.

### Pea Soup.

*Scale per Gallon.*

1¼ lb. Turnips.
2 oz. Pea flour.
1 lb. Onions.
½ lb. Peas.
3 lb. Potatoes.
1 oz. Lentils.
¼ oz. Browning.
Mint, pepper, salt.
½ lb. Shredded cabbage cooked separately, and then mixed in just before serving.
Average cost .43 pence per head.

### Cheese Pudding.

*Scale per Patient.*

4 oz. Bread crumbs.

1 oz. Cheese.

½ oz. Dripping.

1 oz. Onions.

2 oz. Milk.

¼ Teaspoonful egg powder.

⅛ oz. Anchovy sauce.

¼ oz. Parsley.

Pepper, salt.

Average cost 1.18 pence per head.

---

Bread *ad lib.* is also served with all dinners. Pudding is given in addition to soup or broth.

### Vegetable Broth.

*Scale per Gallon.*

1¼ lb. Turnips or carrots.
1 lb. Onions.
2 oz. Pea flour.
4 oz. Haricots.
4 oz. Peas.
3 lb. Potatoes.
1 oz. Lentils.
2 oz. Parsley.
¼ oz. Browning.
½ oz. Yorkshire relish.
½ lb. Shredded cabbage cooked separately, and then mixed in just before serving.
Average cost .52 pence per head.

### Scotch Broth.

*Scale per Gallon.*

1 lb. Onions.
6 oz. Pearl barley.
4 oz. Cheese.
1 oz. Parsley.
1 lb. Parsnips.
8 oz. Swede turnips.
2 lb. Potatoes.
Pepper, salt, mint.
Average cost .49 pence per head.

---

Brown gravy or parsley or other sauces are served with rissoles, cheese pudding, and curry.

